# Updating Phylogeny of Mitochondrial DNA Macrohaplogroup M in India: Dispersal of Modern Human in South Asian Corridor

**DOI:** 10.1371/journal.pone.0007447

**Published:** 2009-10-13

**Authors:** Adimoolam Chandrasekar, Satish Kumar, Jwalapuram Sreenath, Bishwa Nath Sarkar, Bhaskar Pralhad Urade, Sujit Mallick, Syam Sundar Bandopadhyay, Pinuma Barua, Subihra Sankar Barik, Debasish Basu, Uttaravalli Kiran, Prodyot Gangopadhyay, Ramesh Sahani, Bhagavatula Venkata Ravi Prasad, Shampa Gangopadhyay, Gandikota Rama Lakshmi, Rajasekhara Reddy Ravuri, Koneru Padmaja, Pulamaghatta N. Venugopal, Madhu Bala Sharma, Vadlamudi Raghavendra Rao

**Affiliations:** 1 Anthropological Survey of India, Southern Regional Centre, Bogadi, Mysore, India; 2 Anthropological Survey of India, Central Regional Centre, Nagpur, India; 3 Anthropological Survey of India, 27, Kolkata, India; 4 Anthropological Survey of India, Northeast Regional Centre, Shillong, India; 5 Anthropological Survey of India, Andaman & Nicobar Regional Centre, Port Blair, India; Institut Pasteur, France

## Abstract

To construct maternal phylogeny and prehistoric dispersals of modern human being in the Indian sub continent, a diverse subset of 641 complete mitochondrial DNA (mtDNA) genomes belonging to macrohaplogroup M was chosen from a total collection of 2,783 control-region sequences, sampled from 26 selected tribal populations of India. On the basis of complete mtDNA sequencing, we identified 12 new haplogroups - M53 to M64; redefined/ascertained and characterized haplogroups M2, M3, M4, M5, M6, M8′C′Z, M9, M10, M11, M12-G, D, M18, M30, M33, M35, M37, M38, M39, M40, M41, M43, M45 and M49, which were previously described by control and/or coding-region polymorphisms. Our results indicate that the mtDNA lineages reported in the present study (except East Asian lineages M8′C′Z, M9, M10, M11, M12-G, D ) are restricted to Indian region.The deep rooted lineages of macrohaplogroup ‘M’ suggest in-situ origin of these haplogroups in India. Most of these deep rooting lineages are represented by multiple ethnic/linguist groups of India. Hierarchical analysis of molecular variation (AMOVA) shows substantial subdivisions among the tribes of India (Fst = 0.16164). The current Indian mtDNA gene pool was shaped by the initial settlers and was galvanized by minor events of gene flow from the east and west to the restricted zones. Northeast Indian mtDNA pool harbors region specific lineages, other Indian lineages and East Asian lineages. We also suggest the establishment of an East Asian gene in North East India through admixture rather than replacement.

## Introduction

DNA polymorphisms reveal a population's genetic structure, migration and admixture in the past, susceptibility to illness and genetic causes of diseases. A phylogenetic approach is strongly recommended to avoid spurious positive associations between mtDNA mutations and diseases [1]. The pathogenic role of the mitochondrial genome requires more extensive surveys of the mtDNA sequences in different populations and patient groups. Technological improvements in DNA sequencing has made it possible to sequence complete mtDNA genome faster. Attempts have been made to reconstruct the phylogenies and prehistoric dispersal of modern humans in Europe, Africa, Oceania, East Asia, Southeast Asia and South Asia [2], [3]–[24] with complete mtDNA sequence information.

The out-of-Africa scenario [25] has hitherto provided little evidence of the precise route by which modern humans might have left Africa. Two major routes of dispersal have been hypothesized: one is through North Africa into the Levant [26], and another is through Ethiopia along South Asia [27]–[28]. The proposed northern route of initial dispersal of modem humans from Africa could not be sustained by complete and in-depth analysis of mtDNA in recent times [29]. The mitochondrial haplogroup M which was first regarded as an ancient marker of East-Asian origin [30]–[31], had been found at high frequency in India [32] and Ethiopia [33], thus raising the question of its origin. The presence of M haplogroup in Ethiopia, named M1, led to the proposal that haplogroup M originated in eastern Africa, approximately 60,000 years ago, and was carried towards Asia [34]. Contrary to the above, in 2006, Olivieri [35] reported that about 40,000 to 45,000 years ago, predominant North African clades M1 and U6 arose in southwestern Asia and moved together to Africa. Their arrival temporally overlapped the event(s) that led to the peopling of Europe by modern humans and most likely the result of the same change in the climatic conditions that allowed humans to enter in to the Levant, opening the way to the colonization of both Europe and North Africa. In the light of above, the origins of Asian M lineage in Eastern Africa became ambivalent.

Macrohaplogroup M is ubiquitous in India and covers more than 70 per cent of the Indian mtDNA lineages [28], [36]–[38]. Recent studies on complete mtDNA sequences (∼187) tried to resolve the phylogeny of Indian macrohaplogroup M. As a result, M2, M3, M4, M5, M6 [28], [36], [39]–[40], M18, M25 [38], M30, [41], M31 [42], [24] M33, M34, M35, M36, M37, M38, M39, M40 [22], M41, M42 [43], M43 [23], [44], M45 [45], M48, M49, and M50 [46] haplogroups of M that was identified in India helped to a certain extent in understanding M genealogy in diversified Indian populations. In the above background, extensive sequencing of complete mtDNA of South Asia, particularly India, is essential for better understanding of the peopling of the non-African continents, and pathogenesis of diseases in various ethnic groups with different matrilineal backgrounds.

## Results

**Figure 1:**
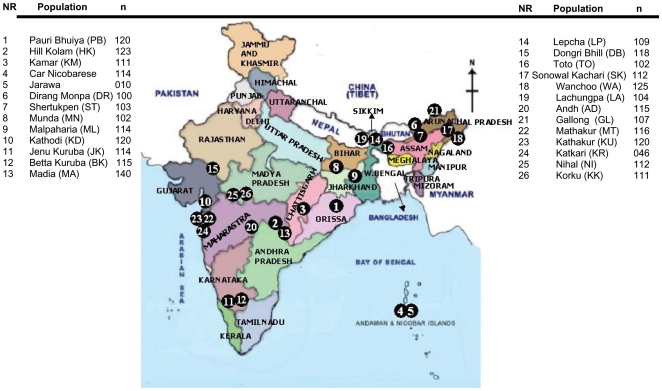
Geographical locations of the Indian tribal populations in the present study.

**Figure 2:**
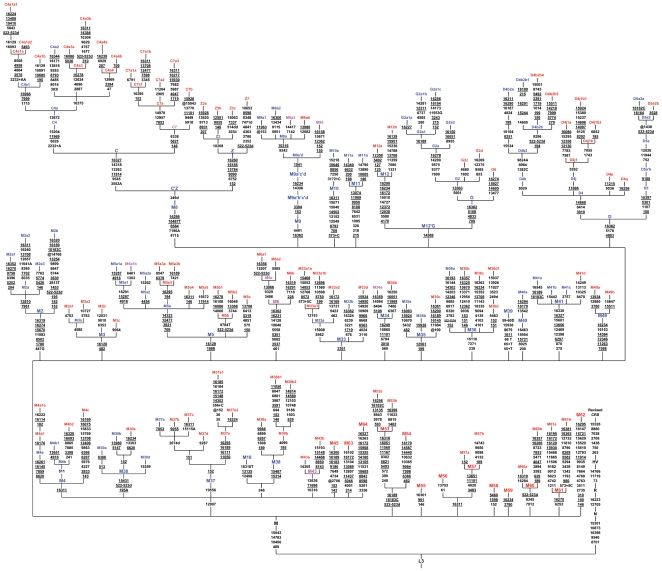
Phylogenetic tree based on complete mtDNA sequences for haplogroup M in India. Suffixes A, C, G, and T indicate transversions, “d” indicates a deletion, and a plus sign (+) indicates an insertion; 9bpins means 9-bp insertion (CCCCCTCTA) in the COII/tRNA^Lys^ intergenic region. The A/C stretch length polymorphism in regions 16180–16193 and 303–315 and mutation 16519, all known to be hyper variable, were disregarded for tree reconstruction; recurrent mutations are underlined and the @ indicates back mutation. Haplogroup names indicated in Blue are defined in the earlier works, pink are redefined and red are newly identified in the present study.

The frequency distribution of M haplogroups has been shown in Table 1. In the present study, 12 novel haplogroups M53 to M64 (Table 2) have been identified, and the phylogenetic status of previously identified haplogroups based on control region and/or coding region information have been ascertained or redefined from 26 tribal population based dataset (Fig. 1). The novel haplogroups are named according to the nomenclature system published elsewhere [47]. Phylogeny tree based on 737 (641 from our study and 96 from earlier studies) complete mtDNA sequences, for haplogroup M in India is shown in Fig. 2.

**Table 1:** Frequency (in Percentage) of haplogroups by populations.

	DR	GL	LA	LP	SK	ST	WA	AD	BK	DB	HK	JK	KD	KK	KM	KR	KU	MA	ML	MN	MT	NI	PB	TT
M2	–	–	–	–	2	–	–	26	64	5.9	29	7.9	26	34	16	31	29	32	13	11	29	14	5.6	–
M3	–	–	–	–	2	–	–	3.6	–	2.9	13	6.8	9.5	–	24	1.9	1.3	2.5	–	–	–	–	–	–
M4	–	–	–	–	2	5.7	10	–	–	2.9	–	–	–	–	–	–	15	1.2	–	–	17	2.5	1.4	–
M5	4.7	–	–	–	–	11	–	18	–	25	3.8	–	1.4	8.9	15	–	2.5	20	4.4	6.3	–	8.2	11	–
M6	–	1.3	–	–	14	–	–	1.8	6.5	–	7.7	–	–	5.3	–	–	–	–	4.4	6.3	–	–	14	–
M8	24	5.1	25	13	4	–	28	–	–	–	–	15	–	–	–	–	–	–	–	–	–	–	–	–
M9	4.7	2.6	6.2	17	14	–	7.5	–	–	–	–	–	–	–	–	–	–	–	–	–	–	–	–	–
M10	–	1.3	–	–	–	–	–	–	–	–	–	–	–	–	–	–	–	–	–	–	–	–	–	–
M11	–	3.8	–	–	–	–	2.5	–	–	–	–	–	–	–	–	–	–	–	–	–	–	–	–	–
M12	–	1.3	–	–	–	–	–	–	–	–	–	–	–	–	–	–	–	–	–	–	–	–	2.8	–
G	2.4	1.3	19	–	–	–	2.5	–	–	–	–	–	1.4	–	–	–	–	–	–	–	–	–	–	–
D	4.7	24	6.2	13	8	40	10	–	–	1.5	–	–	–	–	–	–	–	–	–	–	–	–	–	63
M18	–	–	–	9.9	4	–	–	–	–	1.5	2.6	–	–	5.3	–	–	–	–	29	2.1	–	3.3	1.4	9
M25	2.4	–	–	–	–	–	–	7.3	–	–	13	–	–	–	–	–	–	2.5	–	–	–	–	–	–
M30	–	–	9.4	–	–	–	–	5.5	–	10	–	1.1	5.4	5.3	–	–	2.5	–	–	–	2.4	2.5	–	–
M31	–	–	–	–	–	–	–	–	–	–	–	–	–	–	–	–	–	–	–	4.2	–	–	6.9	–
M33	–	–	–	23	2	–	–	–	–	10	–	–	–	1.8	–	1.9	–	1.2	11	–	–	0.8	–	3
M34	–	–	–	–	–	–	–	–	–	–	–	–	–	–	–	–	–	–	–	2.1	–	0.8	1.4	–
M35	2.4	–	–	–	–	–	–	9.1	8.6	1.5	1.3	–	4.1	–	–	–	–	7.4	–	2.1	–	0.8	–	12
M36	–	–	–	–	–	–	–	–	–	–	–	37	–	–	1	–	–	–	–	–	–	–	–	–
M37	–	1.3	–	–	–	–	–	–	–	5.9	–	–	1.4	–	–	3.7	–	–	–	–	–	2.5	2.8	–
M38	–	–	–	–	–	–	–	–	–	–	–	–	–	3.6	–	3.7	6.3	–	–	4.2	–	–	1.4	–
M39	–	–	–	–	–	–	–	11	5.4	–	–	–	1.4	–	1	–	1.3	2.5	2.2	4.2	–	–	1.4	–
M40	–	3.8	–	–	–	–	–	–	–	–	–	–	–	1.8	1	–	–	–	2.2	15	–	1.6	9.7	–
M41	–	–	–	–	–	–	–	–	–	–	–	–	–	–	2.1	–	–	1.2	8.8	–	–	–	–	–
M43	2.4	–	–	–	–	5.7	–	–	–	–	–	–	–	–	–	–	–	–	–	–	–	–	–	–
M45	–	–	–	–	–	–	–	–	–	–	2.6	–	–	3.6	–	–	–	–	–	8.4	–	–	–	–
M49	26	–	–	–	2	–	2.5	–	–	–	–	–	–	–	–	–	–	–	–	–	–	–	–	–
M53	–	–	–	–	–	–	–	–	–	–	–	–	–	–	11	–	–	–	–	–	–	4.9	5.6	–
M54	–	–	–	–	–	–	–	–	–	–	1.3	29	–	–	–	–	–	–	–	–	–	–	–	–
M55	–	–	–	–	–	–	–	–	–	–	–	–	–	–	–	3.7	–	–	–	–	9.7	–	–	–
M56	–	–	–	–	–	–	–	–	–	–	–	–	–	3.6	–	–	–	–	–	–	–	–	–	–
M57	–	–	–	–	–	–	–	–	–	4.4	–	–	–	–	–	3.7	2.5	–	–	–	–	0.8	–	–
M58	–	–	–	–	–	–	2.5	–	–	–	–	–	–	–	–	–	–	–	–	8.4	–	0.8	–	–
M59	–	–	–	–	4	–	–	–	–	–	–	–	–	–	–	–	–	–	2.2	–	–	–	1.4	–
M60	–	3.8	–	–	4	–	–	–	–	–	–	–	–	–	–	–	–	–	–	–	–	–	–	6
M61	2.4	–	12	–	–	23	–	–	–	–	–	–	–	–	–	–	–	–	–	–	–	–	–	–
M62	4.7	–	–	–	–	–	–	–	–	–	–	–	–	–	–	–	–	–	–	–	–	–	–	–
M63	–	–	–	–	–	–	–	–	–	–	–	–	–	–	–	–	–	7.4	–	–	–	–	–	–
M64	–	–	–	–	–	–	–	–	–	–	–	–	–	–	–	–	–	–	–	–	–	0.8	–	–

**Table 2:** Coding region diagnostic mutations of novel M haplogroups.

Haplogroups	Diagnostic coding region substitutions	Populations
M53	5493–5821–9302–11167–11560	KM, NI, PB & RI88*
M54	9064	JK, HK, MA & AD
M55	961	MT & KR
M56	13753	KK
M57	3483–4020–11101–13651	KU, KR & DB
M58	1598–5460	NI, MN, WA & R58*
M59	3780	PB, SK & ML
M60	7912–8345	GL, SK & TO
M61	6253	DR, ST & LA
M62	2735–3511–4763–7664–8149–9935–11914–12793–15510–15520–15629–15721	DR
M63	4001–5046–11437–12007–12807–13089–13104	MA
M64	3338–5201–8598–8843–10685–11531–13105–14180–15355–15968	NI

*From [22].

### Novel haplogroups in India

The phylogeny trees of haplogroups M53 to M62 have been shown in Fig. S1. Haplogroup M53 encompasses ten samples from Kamar, 6 samples from Nihal, 4 samples from Pauri Bhuiya of the present study and R188 of [22]. Haplogroup M53 has diversified in the central India. Haplogroup M54 is the most frequent in Jenu Kuruba of southern India and insignificant in Hill Kolam of central India. Haplogroup M55 has been identified in one Katkari and eight Mathakur samples from western India. Haplogroups M56 and M57 have been sharing a nucleotide transition at np 16311. Haplogroup M56 has been identified among the Korku of central India. Haplogroup M57 is recognizable among three Dongri Bhill, two Katakari and a Kathakur sample drawn from western India. Haplogroup M58 encompasses 4 Munda samples, 1 Nihal, 1 Wanchoo and R58 of [22]. The earliest branch of this lineage has been represented by Wanchoo tribe in Northeast India, whereas the younger branches are present in central Indian tribes. Haplogroups M63 and M64 have been added to the superbranch, M4′30 nested in M. Six Madia sequences have been grouped into haplogroup M63 (Fig. S2). Haplogroup M64 has been identified in Nihal population of central India (Fig. S2).

### Novel haplogroups in northeast India

Haplogroup M59 encompasses 1 Pauri Bhuiya, 2 Sonowal Kachari, and 1 Malpaharia samples from East and Northeast India. Haplogroup M60 constitutes three samples from the Gallong, two samples from Sonowal Kachari and two samples from Toto, drawn from Northeast Indian tribal populations. The Dirang Monpa (1), Shertukpen (4) and Lachungpa (5) form a new haplogroup named as M61. Lineages DR53 and DR79 from Assam state share eleven specific coding region mutations and seven control region mutations to form a haplogroup, M62 (Fig. S1).

### Refinement of previous haplogroups

A super-branch, M4′30 nested in macrohaplogroup M harbors haplogroups M4, M18, M30, M37, M38, M43, M45, M63 and M64 that shares the transition at np 12007 and encompasses 95 complete mtDNA sequences, represented by all tribal populations except Malpaharia, Sonowal Kachari, Betta Kuruba, Toto and Wanchoo (Fig. S2). From our large dataset, subhaplogroup M2a and M2b of M2 remain unchanged (Fig. S3). Under haplogroup M3, subhaplogroup M3a has been subdivided into M3a1 and M3a2 in the present study and it encompasses 10 Kamar, 4 Kathodi, 6 Jenu Kuruba, 1 Katkari and 1 Dongri Bill samples. The new sub branch M3b encompasses six Kamar sequences from central India. M3c encompasses one sample each from Madia and Andh. (Fig. S3).

A new subhaplogroup, M4c of M4 has been identified in Shertukpen (ST36) and Dirang Monpa (DR77) of Northeast India (Fig. S2). The frequency and diversity of haplogroup M5 reveals that it might have originated in central India and spread out to the eastern and western regions of India. Presence of M5a1b in Slavonic populations [48] and western Indians show its recent migration into the Eurasia. Novel subhaplogroups M5a3 to M5a5 have been defined in the present study while single sequences reported by [22] T13 and A64, B26 have been assigned to M5b and M5c haplogroups respectively (Fig. S4).

Haplogroup M6 has been redefined with 9 mutations, unlike in the earlier study [22] with 11 mutations. The haplogroup M6 has branched into M6a and M6b. M6a has further branched into M6a1 and M6a2 in the present study. The Lineages of Pauri Bhuiya, Munda, Hill Kolam and R56 of [22] have been classified under M6a1. Lineages R65 of [22] and P31 of [23], categorized under M6b earlier, have been assigned to M6a2 in the present study. Subhaplogroup M6b has been found in Korku (KK56) and Andh (AD27) of central India (Fig. S3).

Haplogroups M30, M31, M33, M34, M35, M37, M39, M40, M41, M43, M45 and M49 have been well defined with additional data in the present study. Basal definition of M30 corroborates with earlier works [22]. Subhaplogroup M30e has been newly identified among Kathodi, Kathakur and Mathakur of western region of India (Fig. S2). One of the Andaman's specific haplogroup M31 has been identified in 5 Pauri Bhuiya and 2 Munda samples from our database, and the results have been published [24]. In the present study under Haplogroup M33 from 25 samples of 7 tribes, sub-branches M33a [23], M33b [46] and M33c have been identified. M33a has been further subdivided into 3 new subhaplogroups i.e., M33a1, M33a2 and M33a3. Seven Lepcha (LP) samples have been grouped under M33a1a, whereas 7 samples of Dongri Bhill have been categorized under M33a1b along with T9 sample of [23]. Sample C182 of [22] has been assigned as M33a2 along with Nihal, Korku and Katkari samples. A lineage of Toto has been assigned as M33a3. The lineages of Madia (MA114) and Sonowal Kachari (SK53) have been grouped under M33b. Five samples of Malpaharia have been clustered into a new subhaplogroup M33d (Fig. S6).

M34b, a subhaplogroup of M34 has been newly defined, and another subhaplogroup M34a [23] has been redefined in the present study. Samples MN42 and PB103 have been grouped under M34a. NI37 of the present study along with C56 of [22] formed M34b (Fig. S6). Two new subhaplogroups M35b and M35c have been added to the existing M35 phylogeny tree. Subhaplogroup M35a has been reported from Betta Kuruba (8 samples), Andh (3samples), and a sample each from Nihal, Hill Kolam and Dongri Bhill. M35b encompasses 12 sequences of the study and sequence T17 of [22]. Two samples each from Kathodi and Andh have been categorized under M35c (Fig. S6). M35b, a founder lineage of Roma is present in gene pools of different Slavonic groups (such as Slovaks, Czechs, Poles, and Russians). It provides an evidence of Indian origin of Roma population [48].

In the present study, haplogroup M36 has been classified into 4 subgroups, M36a, M36b, M36c and M36d. This group consists of 33 sequences of Jenu Kuruba and one sequence of Kamar. Both the populations belong to Dravidian groups of South India (Fig. S7). Haplogroup M37 is characterized by mutation at sites 10556 and 152 [22]. Samples of Reddy (R45) and Rathwa (R1) have been classified under M37a [22]–[23]. Haplogroup M37 has been further classified into M37b, M37c, M37d, M37e1 and M37e2 in the present study. The lineages from Nihal and Kathodi have been named as M37b. Three samples of Dongri Bhill have been named as, M37c and two samples of Katkari have been named as M37d. Sample C26 of [22] has been assigned to subhaplogroup, M37e1. Subhaplogroup M37e2 consists of Dongri Bhill (DB110) and Pauri Bhuiya (PB87 and PB89) lineages. A Gallong sample (GL66) shares basal mutations of M37 and has been distinguished as a separate lineage with 14 private mutations (Fig. S2).

Monophyletic origin of M38 and M18 [22] has been confirmed in the present study. Basal mutations of haplogroup M38 remain the same as earlier work [22]. We defined 2 new subhaplogroup of M38 as M38a and M38b. Lineages T72 and A24 of [22] has been reassigned to M38a. Subhaplogroup M38b has been further classified into M38b1 with two Korku and a Pauri Bhuiya lineages, and M38b2 with two Katkari and five Kathakur lineages. Haplogroup M18 has been again redefined in the present study (Fig. S2). Haplogroup M18 has high frequency in Malpaharia tribe (29%). Haplogroup M39 has been identified in 9 tribal populations from central, southern and eastern regions of India (Fig.S7).

Characteristics of M40 haplogroup are similar to the earlier works [22]. Samples T6, R59 of [22] and our 22 samples from seven tribal populations have been grouped under subhaplogroup M40a (Fig. S7). Haplogroup M41 and its sub branches M41a, M41b, M41c have been defined in earlier work [23]. Subhaplogroup M41a has been identified in Malpaharia and M41b in Madia population, whereas Kamar lineages have represented by a new subgroup, M41d in the present study (Fig. S7). M42 has been identified in 1 Pauri Bhuiya, 3 Madia and 3 Munda samples from our database, and the results have been published [43].

Haplogroup M43 has been identified in Dirang Monpa and Shertukpen of Northeast India and has been further classified into M43a and M43b (Fig. S7). Haplogroup M45 of [45] has been redefined in the study. It harbors sequences from Munda, Korku and Hill Kolam tribal populations of central India. Haplogroup M49 has been identified in Bhoi of Meghalaya [46]. In the present study, this haplogroup has been identified in 11 samples of Dirang Monpa, one sample each in Sonowal Kachari and Wanchoo of Northeast India. These Lineages cluster into a new subhaplogroup, M49a, whereas sample BH1 of [46] is assigned to subhaplogroup M49b (Fig. S7).

### East Asian haplogroups in India

It has been interesting to identify major East Asian haplogroups M8′C′Z, M9, M10, M11, M12′G & D in India. East Asian lineages [1], [18], [49] have been identified on the basis of complete mtDNA sequences in the Northeast Indian populations. Several novel sub branches emerged from our study (Fig. S5), thus largely broadening our understanding of human dispersal in South-East Asia.

Haplogroups C&Z are sister subhaplogroups of M8 [50]. Under subhaplogroup C, C4a1 is defined in Han Chinese [1]. In the present work, a new lineage C4a1a has been defined for Lepcha, Lachungpa and Wanchoo populations. The Chinese sample (XJ8435) [1] has been reassigned to C4a1b instead of C4a1. Further, two new lineages, C4a3 and C4a4 have been assigned for Indian samples. Chinese sample (LN7710) [1] and samples of Dirang Monpa, Wanchoo and Gallong have been redefined as the subhaplogroup C7. Eleven Indian samples have been defined as C7a1, C7a2 and Sequence LN7710 of [1] has been reassigned to C7a3. In Gallong population, a new subhaplogroup C7b has been identified. Characterization of Z haplogroup is similar to the earlier work [18]. Four Dirang Monpa sequences have been grouped into a new subgroup Z6. Other Indian samples (Lepcha, Lachungpa and Dirang Monpa) have been named as Z3a while Gallong samples have been grouped under Z3b. Japanese sequence JD21 [18] has been reassigned as Z3c. The largest diversity of sister haplogroup C has been reported in Korea (100%) followed by central Asia (86%), and northern China (78%–74%). Therefore, C can be considered a clade with a Northeast Asian radiation [18]. Representatives of subhaplogroup Z extend from the Saami [4] and Russians [51] of west Eurasia to the people of the eastern peninsula of Kamchatka, the Russian Far East [52]. Its largest diversities are found in Korea (88%), followed by northern China (73%), and central Asia (67%), compatible with the hypothesis of central-east Asian origin of radiation for this haplogroup [18] (Fig. S5).

Haplogroup D has the highest frequency in central and East Asia including Japan. Sub lineages of D, D1, D2 and D3 denote Native American lineages [53]. D4 and D5 have been proposed for Asian lineages [50] whereas D6 has been marked for Japanese. In addition to D4a and D4b, 12 new branches (D4c to D4n) have been defined in Japanese populations [18]. In the present study, subhaplogroups D4b and D4j have been identified in Dirang Monpa, Lepcha, Toto, Wanchoo and Sonowal Kachari. The new sub branches D4p and D4q have been identified in this study. D4p has been identified in Sonowal Kachari and Lachungpa, whereas D4q has been identified in Dirang Monpa, Toto and Shertukpen. Gallong and Shertukpen lineages have genetic linkage with Japanese by sharing D4b2b haplogroup. Haplogroup D4j has been defined by transition at np 11696 [18] and is the most frequent one among the Northeast Indian populations (Toto, Gallong, Lepcha, Lachungpa, Wanchoo and Dirang Monpa) (Fig. S4). Subhaplogroup D5a2 has been identified in Gallong, Sonowal Kachari and Wanchoo of North East India. The geographic distribution of D lineages is peculiar. For example, D5 is prevalent in southern China. D4a is abundant in Chukchi of Northeast Siberia, but D4a1 and D4n have its highest frequency in the Japanese populations [18]. Whereas, D4j is frequent in Northeast Indian populations.

Haplogroup E shares M9 defining mutations [1]. We followed the haplogroup nomenclature of 2009 by [54] for consistency. Indian samples (LA70, LA32, DR46, DR100), a Chinese sample (XJ8420) and a Japanese sample (PD11) are clustered under M9a3. Another 17 Indian samples have been clustered into M9d lineage. M9 has a central and eastern Asian geographic distribution, and it has reached its greatest frequency (11%) in Tibet. Present Indian samples, which consist of halopgroup M9, are geographically adjacent to Tibet. In addition to mainland Japanese, M9 has been detected in the indigenous Ainu and Ryukyuans [55] (Fig. S5). Haplogroup M10a [1] has been identified in Gallong population. Although its highest frequency is among Tibetans (8%), rich diversity is found in China. It is present among Koreans and mainland Japanese, but has not been detected in either Ainu or Ryukyuans [18] (Fig. S5). In the present study, M11a has been redefined and assigned to Chinese, whereas M11b has been assigned to Japanese [18]. Indian samples (GL19, GL80, GL88, and WA94), clustered under M11a, indicate a genetic affinity with East Asians (Fig. S5). Mutation at np15924 found at the root of M11 and M12 in Japanese [18], has been absent both in Indian and Chinese samples.

In the present study, haplogroup M12′G defining mutation site (at np 14569) is similar to the definition in earlier work [1]. Sample GD 7825 of [11] and our sample GL31 have been assigned to a new subgroup, M12a. Samples of Pauri Bhuiya (PB8 and PB119) have been defined as a new group M12b. Subgroup G2a1a which is present among Japanese has been identified in Wanchoo and Lachungpa populations. Novel subhaplogroups G2c, G2d and G6 have been defined in the present study. One lineage each of Gallong (GL61) and Kathodi (KD106) form subhaplogroup G2c. Subhaplogroup G2d harbors Lachungpa samples. G6 has been found in Lachungpa and Dirangmonpa populations. The frequency distribution of G2 is abundant in northern China and central Asia, reaching higher frequencies in the southern Siberia. Clades G3 and G4 have been apparent in Japanese. Subgroup G5 is dominant in northeastern Siberia. However, G1a1 has the highest frequencies in a cluster embracing Japanese and Koreans [18] (Fig. S5).

### Age estimates

The age estimates of the M haplogroup using coding region mutation rate (1.26±0.08×10^−8^) [12] have been listed in Table 3. Indian specific haplogroups of M - M2, M6, M38, M53, M54, M58, M59, M62, and East Asian specific haplogroups M8, M11, M12′G in India are rather ancient with ages >50,000 years. Whereas, haplogroups M3, M30, M37 have younger founder ages, i.e.<25,000 years. The ages of the remaining haplogroups range from 26,000–50,000 years. The Indian M haplogroup founder age has been estimated as 66,000±9,000 years. The coalescence age of East Asian M lineages in Northeast India (69,000±7,000 years) is similar to the East Asian (69,000±5000 years by [11]) age.

**Table 3:** Diversity and age estimates for M haplogroups in India.

Haplogroup	n	ρ	σ	TMRCA (yrs)	SD	Founder age (yrs)^1^	SD	TMRCA (yrs)	SD	Founder age (yrs)^2^	SD
M2a	39	6.49	1.5	33,000	8,000			36,000	9,000		
M2b	21	1.62	0.81	8,000	4,000			10000	5,000		
**M2 Total**	**60**	**8.53**	**1.65**	**44,000**	**8,000**	**64,000**	**13,000**	**39,000**	**10000**	**52,000**	**14,000**
M3a	25	3.8	1.08	20,000	6,000			17,000	9,000		
M3b	6	0.67	0.44	3,000	3,000			5,000	5,000		
**M3 Total**	**34**	**4.53**	**1.14**	**23,000**	**6,000**	**23,000**	**6,000**	**21,000**	**7,000**	**21,000**	**7,000**
M4a	4	2.25	0.75	12,000	4,000			7,000	3,000		
M4b	7	6.57	1.47	34,000	8,000			26,000	8,000		
M4c	2	0.5	0.5	3000	3,000			3,000	3,000		
**M 4 Total**	**13**	**6.46**	**1.05**	**33,000**	**5,000**	**33,000**	**5,000**	**23,000**	**5,000**	**23,000**	**5,000**
M5a	49	3.22	0.74	17,000	4,000			13,000	6,000		
M5b	12	7.08	1.58	36,000	8,000			37,000	10,000		
M5c	3	5.67	1.73	29,000	9,000			18,000	7,000		
**M5 Total**	**64**	**7.5**	**1.67**	**39,000**	**9,000**	**44,000**	**10,000**	**36,000**	**10,000**	**36,000**	**10,000**
M6a	10	2.6	0.94	13,000	5,000			12,000	6,000		
M6b	2	0.5	0.5	3,000	3,000			3,000	3,000		
**M6 Total**	**12**	**4.92**	**1.65**	**25,000**	**8,000**	**56,000**	**14,000**	**23,000**	**9,000**	**43,000**	**15,000**
C	42	9.33	1.74	48,000	9,000			31,000	8,000		
Z	16	8.75	1.86	45,000	10,000			40,000	11,000		
**M8**′**C**′**Z Total**	**58**	**13.62**	**2.17**	**70,000**	**11,000**	**91,000**	**15,000**	**58,000**	**12,000**	**79,000**	**17,000**
M9a	17	3.24	1.03	17,000	5,000			13,000	5,000		
M9d	9	5.33	1.59	27,000	8,000			12,000	5,000		
**M9Total**	**26**	**5.62**	**1.31**	**29,000**	**7,000**	**49,000**	**12,000**	**15,000**	**4,000**	**22,000**	**8,000**
**M10**	**3**	**8**	**2.36**	**41,000**	**12,000**	**82,000**	**19,000**	**34,000**	**13,000**	**61,000**	**19,000**
M11a	6	2.33	0.67	12,000	3,000			9,000	3,000		
M11b	2	2.5	1.12	13,000	6,000			3,000	3,000		
**M11 Total**	**8**	**4.63**	**1.28**	**24,000**	**7,000**	**60,000**	**15,000**	**16,000**	**6,000**	**43,000**	**15,000**
M12	4	7.5	1.73	39,000	9,000			25,000	8,000		
G	16	8.5	1.68	44,000	9,000			38,000	9,000		
**M12**′**G Total**	**20**	**11.9**	**2.02**	**61,000**	**10,000**	**66,000**	**12,000**	**43,000**	**10,000**	**50,000**	**12,000**
D4	58	6.22	1.25	32,000	6,000			22,000	6,000		
D5	12	7.58	2.16	39,000	11,000			25,000	10,000		
**D Total**	**70**	**9.46**	**3.36**	**49,000**	**9,000**	**59,000**	**12,000**	**30,000**	**8,000**	**36,000**	**10,000**
**M18**	**4**	**5**	**1.12**	**26,000**	**6,000**	**34,000**	**9,000**	**7,000**	**3,000**	**14,000**	**8,000**
**M30 Total**	**32**	**3**	**0.5**	**15,000**	**3,000**	**20,000**	**6,000**	**9,000**	**3,000**	**9,000**	**3,000**
M33a	20	6.25	1.47	32,000	8,000			25,000	7,000		
M33b	4	8.5	1.9	44,000	10,000			42,000	11,000		
**M33 Total**	**29**	**8.83**	**1.54**	**45,000**	**8,000**	**51,000**	**9,000**	**29,000**	**6,000**	**29,000**	**6,000**
M34a	3	3.33	1.05	17,000	5,000			18,000	6,000		
M34b	2	1	0.71	5,000	4,000			7,000	5,000		
**M34 Total**	**5**	**5.4**	**1.43**	**28,000**	**7,000**	**48,000**	**13,000**	**28,000**	**9,000**	**49,000**	**16,000**
M35a	17	2.47	0.78	13,000	4,000			16,000	7,000		
M35b	14	4.36	0.97	22,000	5,000			18,000	5,000		
M35c	4	3	1.22	15,000	6,000			10,000	6,000		
**M35 Total**	**35**	**5.26**	**1.09**	**27,000**	**6,000**	**32,000**	**8,000**	**23,000**	**6,000**	**30,000**	**9,000**
M36d	27	5.3	1.98	27,000	10,000			16,000	9,000		
**M36 Total**	**36**	**6.69**	**1.76**	**34,000**	**9,000**	**45,000**	**12,000**	**26,000**	**9,000**	**33,000**	**11,000**
M37abcd	10	1.7	0.12	8,736	4,000			5,000	3,000		
M37e	4	1.5	0.71	8000	4,000			8,000	4,000		
**M37 Total**	**15**	**2.6**	**0.61**	**13,000**	**3,000**	**19,000**	**6,000**	**10,000**	**3,000**	**17,000**	**7,000**
M38a	2	3.5	1.32	18,000	7,000			14,000	7,000		
M38b	12	10.3	2.31	53,000	12,000			47,000	12,000		
**M38 Total**	**14**	**10.67**	**2.14**	**55,000**	**11,000**	**65,000**	**13,000**	**42,000**	**10,000**	**49,000**	**12,000**
**M39 Total**	**24**	**3.96**	**0.89**	**20000**	**5,000**	**36,000**	**10,000**	**11,000**	**3,000**	**17,000**	**8,000**
**M40**	**24**	**3.83**	**1.15**	**20,000**	**6,000**	**35,000**	**11,000**	**17,000**	**7,000**	**31,000**	**12,000**
**M41**	**10**	**1.8**	**0.51**	**9,000**	**3,000**	**40,000**	**13,000**	**8,000**	**3,000**	**28,000**	**12,000**
**M43**	**4**	**6.5**	**1.58**	**33,000**	**8,000**	**49,000**	**12,000**	**25,000**	**8,000**	**39,000**	**13,000**
**M45**	**8**	**2.63**	**0.72**	**13,000**	**4,000**	**29,000**	**10,000**	**14,000**	**5,000**	**28,000**	**11,000**
**M49**	**14**	**4.79**	**2.02**	**25000**	**10,000**	**45,000**	**15,000**	**13,000**	**8,000**	**20,000**	**11,000**
**M53**	**22**	**4.95**	**1.83**	**25,000**	**9,000**	**56,000**	**16,000**	**17,000**	**9,000**	**38,000**	**15,000**
**M54 Total**	**29**	**2.19**	**1.10**	**11,000**	**6,000**	**47,000**	**15000**	**26,000**	**12,000**	**26,000**	**12,000**
**M55 Total**	**10**	**4.1**	**1.51**	**21,000**	**8,000**	**26,000**	**9,000**	**11,000**	**6,000**	**11,000**	**6,000**
**M56 Total**	**2**	**3**	**1.22**	**15,000**	**6,000**	**21,000**	**8,000**	**20,000**	**8,000**	**20,800**	**8,000**
M57a	3	7.67	2.03	39,000	10,000			32,000	11,000		
M57b	3	0.67	0.58	3,000	3,000			8,000	3,000		
**M57 Total**	**8**	**8.75**	**1.87**	**45,000**	**10,000**	**66,000**	**14,000**	**25,000**	**7,000**	**45,000**	**14,000**
**M58 Total**	**7**	**9.43**	**2.31**	**48,000**	**12,000**	**59,000**	**14,000**	**29,000**	**11,000**	**29,000**	**11,000**
**M59 Total**	**4**	**7.25**	**1.79**	**37,000**	**9,000**	**42,000**	**11,000**	**30,000**	**10,000**	**37,000**	**12,000**
M60a	5	3.4	1.04	17,000	5,000			12,000	6,000		
M60b	2	1	0.71	5,000	4,000			3,000	3,000		
**M60 Total**	**7**	**4.71**	**1.08**	**24,000**	**6,000**	**35,000**	**9,000**	**15,000**	**5,000**	**22,000**	**9,000**
M61a	5	2	1.17	10,000	6,000			7,000	6,000		
M61b	4	1.5	0.79	8,000	4,000			2,000	2,000		
**M61 Total**	**9**	**10.33**	**2.21**	**53,000**	**11,000**	**58,000**	**12,000**	**38,000**	**11,000**	**38,000**	**11,000**
**M62**	**2**	**1.5**	**0.87**	**8,000**	**4,000**	**69,000**	**18,000**	**7,000**	**5,000**	**68,000**	**21,000**
**M63**	**6**	**6.14**	**2.08**	**32,000**	**11,000**	**37,000**	**12,000**	**1,000**	**1,000**	**28,000**	**14,000**
**Total M In India**	**726**	**9.92**	**0.53**	**51,000**	**3,000**	**66,000**	**9,000**	**36,000**	**6,000**	**56,000**	**3,000**

1 Based on coding-region mutation rate 1.26±0.08×10^−8^.

2 Based on protein-coding synonymous changes of 3.5×10^−8^.

Coalescence time of macrohaplogroup M in India has been estimated using synonymous mutation rate (3.5×10^−8^) [56] which is (36,000±3,000 years) less than the estimate (46,000±5,000 years) by [56] for M haplogroup in Asia.

The total rho estimate for haplogroup M is 9.9±0.5 (Table 3). It includes all the Indian lineages and also East Asian lineages. After excluding the East Asian lineages (M8′C′Z, M9, M10, M11, M12-G, D), the total diversity estimate for haplogroup M in India is 8.7±0.5. It is similar to the earlier works, i.e., 8.7±0.6 [22].

Recurrent mutations generated in each network are summarized in Table S1. Total number of variables sites in the present study is 1092. Out of 1092 variable sites, 270 (24%) had mutated more than once. Of 269 sites, 15 sites mutated 4 or more times. Eleven hotspots were reported in [2], [56] and 3 hotspots (non-synonymous) are reported in the present study.

## Discussion

The Indian mtDNA phylogeny (Fig. 2) has been constructed based on 641 complete sequences of our study and 96 {48 [22], 18 [18], 9 [11], 9 [23], [44], 4 [56], 2 [3], 3 [46], 1 [2] and 2 [48]} from published sequences. It reveals extensive maternal variations emerging from the largest number of deeply rooted autochthonous lineages, reflecting the diversity of populations residing in the sub-continent, who are biologically and culturally distinct. Hierarchical analysis of molecular variations show significant differentiations (Fst = 0.16164) and sub divisions among the populations, with a large fraction of the variance found within populations (83%) (Table 4). Individual population contribution to the global Fst measure has not deviated much from the average (ranges 15–17 per cent), indicating that the degree of evolution of all the populations from a common ancestral population is similar, without any special evolutionary constraints. The problems faced by the earlier work [22] in constructing the Indian mtDNA phylogeny tree with 70 sequences, have been resolved to some extent in this study. For example, the monophyly of M18′38 or the actual placement of the branch referred to as M4a within M4 has been confirmed. In the present study, coding region mutations have been considered for assigning new haplogroups, as hyper variable control region sites lead to confusing conclusions, which are evident from global mtDNA phylogeny, based on complete mtDNA sequences. In this study, 12 novel haplogroups and 25 already defined haplogroups clearly outnumber the basal variation of macrohaplogroup M in any region of the globe.

**Table 4:** Analysis of molecular variance of the tribes in India.

Source of variation	d.f.	Sum of squares	Variance	Percentage of variation
Among populations	23	1675.186	2.31028*	16.16
Within populations	617	7393.209	11.98251	83.84
Total	640	9068.395	14.29279	

Fixation Index Fst: 0.16164*.

*P-value  = 0.00000±0.00000.

The haplogroup M frequency ranges from 50 per cent in Kathodi, Katkari and Gallong to 97 per cent in Jenu Kuruba with an average frequency of 70 percent, which has been consistent with earlier works [28], [32], [37], [39], [57]–[62]. The haplogroup M has high frequency in India and drops abruptly to about 5% in Iran, marking the western border of the haplogroup M distribution [37]. The maternal gene flow in and out of India has been limited since the initial settling of Indian maternal lineages. An eastern and western Eurasian lineage ranges from 10–12 percent in India [37]. Low frequencies of western Eurasian haplogroups in India [32], [38], [63] must have had a recent entry date [<10 thousand years ago] [28]. Tibeto-Burman speaking tribal populations of eastern and northern India exhibits fair frequencies of East Eurasian-specific mtDNA haplogroups, reaching a peak of nearly 50% in the Kanet of Himachal Pradesh [37]. In the present study, fair frequencies of eastern Asian haplogroups were observed in the North East Indian populations (Table 1). The current Indian gene pool has been reshaped *in situ* after initial mtDNA pool was established and galvanized by relatively minor events of gene flow from the West and from the East into India through admixture.

The Indian mtDNA pool consists of several deep-rooted lineages of macrohaplogroup ‘M’ suggesting *in situ* origin [22]–[23], [36]–[37]. It is apparent that all the ancient lineages under analysis emerge directly from the root of the macrohaplogroup M. The phylogenetic status of previously identified haplogroups M2, M3, M4, M5, M6, M18, M30, M33–M41, M43, M45, M48–M50 and the newly identified M53–M64 confined to the Indian subcontinent.

### Macrohaplogroup M lineages in Northeast India

Studies, which focused on Northeast Indian populations largely, concluded that they differ from mainland populations and show affinity to the Southeast Asian populations. But the present study identified Indian mtDNA lineages like M2, M3, M4, M5, M6, M18, M25, M30, M33, M35, M37, M40, M43, M58 and M59, region specific lineages like M49, M60, M61, M62 and East Asian specific lineages like M8′CZ, M9, M10, M11, M12′G and D lineages in northeast Indian populations. It supports modern human habitation in Northeast India during Paleolithic times and genetic continuity between India and East/Southeast Asia. East Asian phylogenetic trees have been broadened in the present study with additional Northeast Indian data. For example Seq XJ8435 [1] of C4a1 has been further assigned to C4a1b and Indian samples (LA50, LA61, LP67, WA46 and WA105) have been classified into C4a1a. Apart from C4a1 and C4a2 of East Asian Phylogeny tree, C4a3 and C4a4 have been defined in the present study. Northeast Indian tribes, particularly Tibeto-Burman linguistic groups indicate genetic affiliation with East Asians. This is in agreement with the earlier works: mtDNA evidence [64]–[65], Y chromosome evidence [66]–[67] and linguistic evidence, [68]. In Northeast India, D4b2b, D4j, D5a2, C4a, C7, M9a, M10a, M11a, M12 and G2a1a haplogroups have the resultant of Last Glacial Maximum (about 20,000 years ago) migrations from southern China and is admixed with local initial settlers.

### Origin of Macrohaplogroup M

L3 lineages other than M and N are absent in India and among non-African mitochondria in general [2]–[3], [49]. M, N and R haplogroups of mtDNA have no indication of an African origin. However, it is proposed that the origin of haplogroup M is in Africa [34], in view of its high frequency in Ethiopia. But in 2006, by [35] demonstrated that the presence of M1 and U6 in Africa is due to a back migration. Sequencing of 81 entire human mitochondrial DNAs belonging to haplogroups M1 and U6 revealed that these predominantly North African Clades arose in Southwestern Asia and moved together to Africa about 40,000 to 45,000 years ago. Only some sub-sets of M1a (with an estimated coalescence time of 28.8±4.9ky), U6a2 (with an estimated coalescence time of 24.0±7.3ky), and U6d (with an estimated coalescence time of 20.6±7.3ky) diffused to East and North Africa through the Levant, leaving the origin of macrohaplogroup M unresolved. Haplogroup M has been found ubiquitous in India, although its frequency is somewhat higher in southern Indian populations than in northern Indian populations and to a large extent autochthonous because neither the East nor the West Eurasian mtDNA pools include such lineages at notable frequencies [37], [58]. Our findings, (for example, deep time depth >50,000 years of western, central, southern and eastern Indian haplogroups M2, M38, M54, M58, M33, M6, M61, M62 and distribution of macrohaplogroup M) do not rule out the possibility of macrohaplogroup M arising in Indian population.

### Migration routes of modern human

Recent mtDNA evidence on modern human out of Africa migration route suggests a single dispersal by a southern coastal route to India and further, to East Asia and Australia [17], [20], [22], [23], [66], [69]. The North Asian route could not get support from mtDNA due to the lack of basal M, R, N lineages in northern Asians, thereby ruling out the existence of a northern Asian route [29]–[30], [70]–[71]. Proven back migration of sub lineages of M and U into Africa [35], and the absence of L3 lineages or ancestral lineage for L3, M and N in India, leaves two issues unresolved: evidences for the southern route hypothesis from India and origin of M haplogroup. However, in the present study, the basal diversity (37 nodes) and founder ages (57,000–75,000 years) of macrohaplogroup M in India reveals initial settlement of African exodus in India. Our database also reveals evidences that Andaman islanders and Australians have ancestral maternal roots in India [24], [43].

In summary, the present study provides evidence that several Indian mtDNA M lineages are deep rooted and *in situ* origin. In North East India the coalescent time of East Asian lineages dates back to Last Glacial Maximum (LGM). Further, the combination of virtually all previously reported lineages from South and East Asia and our newly produced Indian complete mtDNA sequences have helped to define several novel (sub) haplogroups. The present work further ascertained previously reported haplogroups, and refined the phylogenetic tree of South Asia. This updated phylogenetic tree provides an essential reference guide for diseases, anthropological and forensic studies among Asian populations.

## Methods

The Indian populations are organized into 4365 communities [72], which include selfdefined castes, tribes and religious groups. About 450 tribes constitute 8.08% (2001 census) of the total Indian population. They speak more than 750 dialects [73], which can be broadly classified into Austro-Asiatic, Dravidian, Tibeto-Burman and Indo-European language families. The tribes are endogamous in nature and socio-culturally distinct. They inhabit mostly in the forests and hilly terrain areas. Government of India has notified 75 tribes as the most primitive group among the original inhabitants of India. Out of 75 primitive tribal groups, Anthropological Survey of India has selected 26 tribes inhabiting the western, central, southern and eastern parts of India, representing 4 major linguistic families, namely Dravidian, Indo-European, Austro-Asiatic and Tibeto-Burman and collected 2,783 blood samples for the present study Fig.1.

The Ethical Committee of the Anthropological Survey of India approved the project. 5–10 ml of blood was drawn from healthy and unrelated individuals after obtaining written consent. Samples were collected in Vacutainer as per standard protocols, and extraction of DNA was performed according to the enzymatic extraction procedure followed by phenol purification [74], which was standardised at Anthropological Survey of India, C.R.C. laboratory, Nagpur. Out of the total 2,783 samples, M haplogroup samples (1751) were differentiated based on their specific coding region mutations. Among 1751, distinct haplogroup status was ascertained for 654 samples, where as 1097 samples remained as M* samples. Approximately 5 samples of each distinct haplogroups among the 654 amounting to 220 and all 530 M*samples, total 750 were selected for complete sequencing. After checking the quality of sequences, 12 ambiguity sequences removed from final analysis. 641 complete mtDNA sequences were included in the final analysis for the present study and the results of 97 sequences published elsewhere [24], [43], [75]. Complete sequencing was done using 24 pairs of both forward and reverse primers [76]. Sequences were assembled, and edited using SeqScape 2.5. Mutations were scored relative to the revised Cambridge Reference sequence [77]. Deviations from the rCRS were confirmed by manual checking of their electropherograms. Phylogenetic relationships among the sequences were determined by Median-joining net work analysis with the help of Networking 4.1 software. Most parsimonious trees of the mtDNA haplogroups were reconstructed manually following a parsimony approach, and confirmed by the program Networking 4.1. The founder ages and time of TMRCA have been calculated as implemented in [21]. The age of the founder mtDNA type has yielded a time estimate for its arrival in the continent. It includes the ancestral nodes that were shared by its variants in the tree. The ages of haplogroups M are estimated from 736 lineages based on mutation rate 1.26±0.08×10^−8^ [12]. The ages also calculated by using substitution rate estimate for protein-coding synonymous change of 3.5×10^−8^ [56] manually using Rho estimate [53]. The variance of Rho was estimated [78] for both the methods. Nevertheless, all ages calculated without evidence to sustain the assumption of the molecular clock mean that estimation of the associated error values [78] is only an approximation. AMOVA was performed to evaluate the amount of genetic structure among the tribal population using Arlequin var 3.11 [79].

### Quality Control

Out of 1751 M samples, 750 samples were selected for complete mtDNA sequencing. Sequence reactions were carried out with a BigDye terminator cycle sequencing FS ready reaction kit (Applied Biosystems) to produce even signal intensities and to reduce false negatives. It enabled more accurate automated mixed base identification. Sequencing data that were generated on Applied Biosystems 3730 DNA analyzer were analyzed in SeqScape software V 2.5. KB base caller V 1.4 was used in the analysis protocol. KB base caller process florescence signal assigns a base to each peak and assigns quality value (QV) to each base. The QV predicts the probability of a base call error. KB base caller generated QV from 1 to 99. Typically high quality pure bases will have QV ranging from 20–50 (Probability of Error is 1% to 0.001%). Mixed bases were identified if the secondary peak height threshold value was >25%. To set clear range of the sequence quality value method (Remove base from the ends until fewer than 4 bases out of 20 have QVs<20) was used. Filter setting values used were: Maximum mixed bases = 20, Minimum sample score = 25. Depending on the sequence quality and the criteria specified for filtering the data prior to assembly, the samples were not assembled. These unassembled samples were re-sequenced until it satisfied the quality. Editing of data and scoring of mutations were done by two independent groups of researchers. Phylogenetic network was performed and some errors were identified (mixing of contigs etc). 12 Unresolved samples, ambiguity sequences, low quality sequences, error sequences were eliminated for final analysis. 641 complete mtDNA sequences were included in the final analysis for the present study and the results of 97 sequences published elsewhere [24], [43], [75]. To check the reliability of the data, we calculated and compared the diversities with the earlier work [22]. The diversity values corroborated with the earlier work. Further, to ascertain the quality of the results, recurrent mutations generated by the individuals' tree networks were summarized and considering the work by [52] as a reference point, hotspots were rechecked.

All the sequences have been deposited in the NCBI database (Accession Numbers: FJ 383814 to FJ 383174).

### Post script

#### Haplogroup nomenclature conflict

Global mtDNA tree at http://www.phylotree.org presented previously published as well as newly identified haplogroups M51 and M52 in the study [54]. While our paper is under review another study [80] defined haplogroups M51, M52, M53. Whereas M53 name was given to the already defined M45. Thus nomenclature conflict exists between the two studies. Haplogroups M51 and M52 of [80] coincide with our M54 and M58 respectively. We followed mtDNA tree at http://www.phylotree.org and named our new haplogroups from M53 to M64.

## Supporting Information

Table S1 Showing recurrence of mutations at various nucleotide positions (np).
(4.33 MB TIF)

Figure S1 Indian mtDNA phylogenetic tree of macrohaplogroup M. Suffixes A, C, G, and T indicate transversions, “d” indicates a deletion, and a plus sign (+) indicates an insertion; 9bpins means 9-bp insertion (CCCCCTCTA) in the COII/tRNALys intergenic region. The A/C stretch length polymorphism in regions 16180–16193 and 303–315 and mutation 16519, all known to be hyper variable, were disregarded for tree reconstruction; recurrent mutations are underlined and the @ indicates back mutation. Samples code names were given in fig. 1.Samples collected from published sources were referred by symbols SU [22], TK [18], KG [11], TG [23], [43], KS [52], IG [3], BM [44], HE [2] and MC [48] followed by “#” and the original sample code. Haplogroup names indicated in Blue are defined in the earlier works, pink are redefined and red are newly identified in the present study. Coalescence times are based on synonymous mutation rate 3.5X10 ^-8 [52].
(3.76 MB TIF)

Figure S2 Indian mtDNA phylogenetic tree of macrohaplogroup M. Suffixes A, C, G, and T indicate transversions, “d” indicates a deletion, and a plus sign (+) indicates an insertion; 9bpins means 9-bp insertion (CCCCCTCTA) in the COII/tRNALys intergenic region. The A/C stretch length polymorphism in regions 16180–16193 and 303–315 and mutation 16519, all known to be hyper variable, were disregarded for tree reconstruction; recurrent mutations are underlined and the @ indicates back mutation. Samples code names were given in fig. 1.Samples collected from published sources were referred by symbols SU [22], TK [18], KG [11], TG [23], [43], KS [52], IG [3], BM [44], HE [2] and MC [48] followed by “#” and the original sample code. Haplogroup names indicated in Blue are defined in the earlier works, pink are redefined and red are newly identified in the present study. Coalescence times are based on synonymous mutation rate 3.5X10 ^-8 [52].
(3.85 MB TIF)

Figure S3 Indian mtDNA phylogenetic tree of macrohaplogroup M. Suffixes A, C, G, and T indicate transversions, “d” indicates a deletion, and a plus sign (+) indicates an insertion; 9bpins means 9-bp insertion (CCCCCTCTA) in the COII/tRNALys intergenic region. The A/C stretch length polymorphism in regions 16180–16193 and 303–315 and mutation 16519, all known to be hyper variable, were disregarded for tree reconstruction; recurrent mutations are underlined and the @ indicates back mutation. Samples code names were given in fig. 1.Samples collected from published sources were referred by symbols SU [22], TK [18], KG [11], TG [23], [43], KS [52], IG [3], BM [44], HE [2] and MC [48] followed by “#” and the original sample code. Haplogroup names indicated in Blue are defined in the earlier works, pink are redefined and red are newly identified in the present study. Coalescence times are based on synonymous mutation rate 3.5X10 ^-8 [52].
(3.69 MB TIF)

Figure S4 Indian mtDNA phylogenetic tree of macrohaplogroup M. Suffixes A, C, G, and T indicate transversions, “d” indicates a deletion, and a plus sign (+) indicates an insertion; 9bpins means 9-bp insertion (CCCCCTCTA) in the COII/tRNALys intergenic region. The A/C stretch length polymorphism in regions 16180–16193 and 303–315 and mutation 16519, all known to be hyper variable, were disregarded for tree reconstruction; recurrent mutations are underlined and the @ indicates back mutation. Samples code names were given in fig. 1.Samples collected from published sources were referred by symbols SU [22], TK [18], KG [11], TG [23], [43], KS [52], IG [3], BM [44], HE [2] and MC [48] followed by “#” and the original sample code. Haplogroup names indicated in Blue are defined in the earlier works, pink are redefined and red are newly identified in the present study. Coalescence times are based on synonymous mutation rate 3.5X10 ^-8 [52].
(3.94 MB TIF)

Figure S5 Indian mtDNA phylogenetic tree of macrohaplogroup M. Suffixes A, C, G, and T indicate transversions, “d” indicates a deletion, and a plus sign (+) indicates an insertion; 9bpins means 9-bp insertion (CCCCCTCTA) in the COII/tRNALys intergenic region. The A/C stretch length polymorphism in regions 16180–16193 and 303–315 and mutation 16519, all known to be hyper variable, were disregarded for tree reconstruction; recurrent mutations are underlined and the @ indicates back mutation. Samples code names were given in fig. 1.Samples collected from published sources were referred by symbols SU [22], TK [18], KG [11], TG [23], [43], KS [52], IG [3], BM [44], HE [2] and MC [48] followed by “#” and the original sample code. Haplogroup names indicated in Blue are defined in the earlier works, pink are redefined and red are newly identified in the present study. Coalescence times are based on synonymous mutation rate 3.5X10 ^-8 [52].
(3.84 MB TIF)

Figure S6 Indian mtDNA phylogenetic tree of macrohaplogroup M. Suffixes A, C, G, and T indicate transversions, “d” indicates a deletion, and a plus sign (+) indicates an insertion; 9bpins means 9-bp insertion (CCCCCTCTA) in the COII/tRNALys intergenic region. The A/C stretch length polymorphism in regions 16180–16193 and 303–315 and mutation 16519, all known to be hyper variable, were disregarded for tree reconstruction; recurrent mutations are underlined and the @ indicates back mutation. Samples code names were given in fig. 1.Samples collected from published sources were referred by symbols SU [22], TK [18], KG [11], TG [23], [43], KS [52], IG [3], BM [44], HE [2] and MC [48] followed by “#” and the original sample code. Haplogroup names indicated in Blue are defined in the earlier works, pink are redefined and red are newly identified in the present study. Coalescence times are based on synonymous mutation rate 3.5X10 ^-8 [52].
(3.57 MB TIF)

Figure S7 Indian mtDNA phylogenetic tree of macrohaplogroup M. Suffixes A, C, G, and T indicate transversions, “d” indicates a deletion, and a plus sign (+) indicates an insertion; 9bpins means 9-bp insertion (CCCCCTCTA) in the COII/tRNALys intergenic region. The A/C stretch length polymorphism in regions 16180–16193 and 303–315 and mutation 16519, all known to be hyper variable, were disregarded for tree reconstruction; recurrent mutations are underlined and the @ indicates back mutation. Samples code names were given in fig. 1.Samples collected from published sources were referred by symbols SU [22], TK [18], KG [11], TG [23], [43], KS [52], IG [3], BM [44], HE [2] and MC [48] followed by “#” and the original sample code. Haplogroup names indicated in Blue are defined in the earlier works, pink are redefined and red are newly identified in the present study. Coalescence times are based on synonymous mutation rate 3.5X10 ^-8 [52].
(3.59 MB TIF)
