# Supplementary figures and images for: Updating Phylogeny of Mitochondrial DNA Macrohaplogroup M in India: Dispersal of Modern Human in South Asian Corridor
Source: PLoS One. 2009 Oct 13;4(10):e7447. doi: 10.1371/journal.pone.0007447 (PMC2757894; doi:10.1371/journal.pone.0007447)

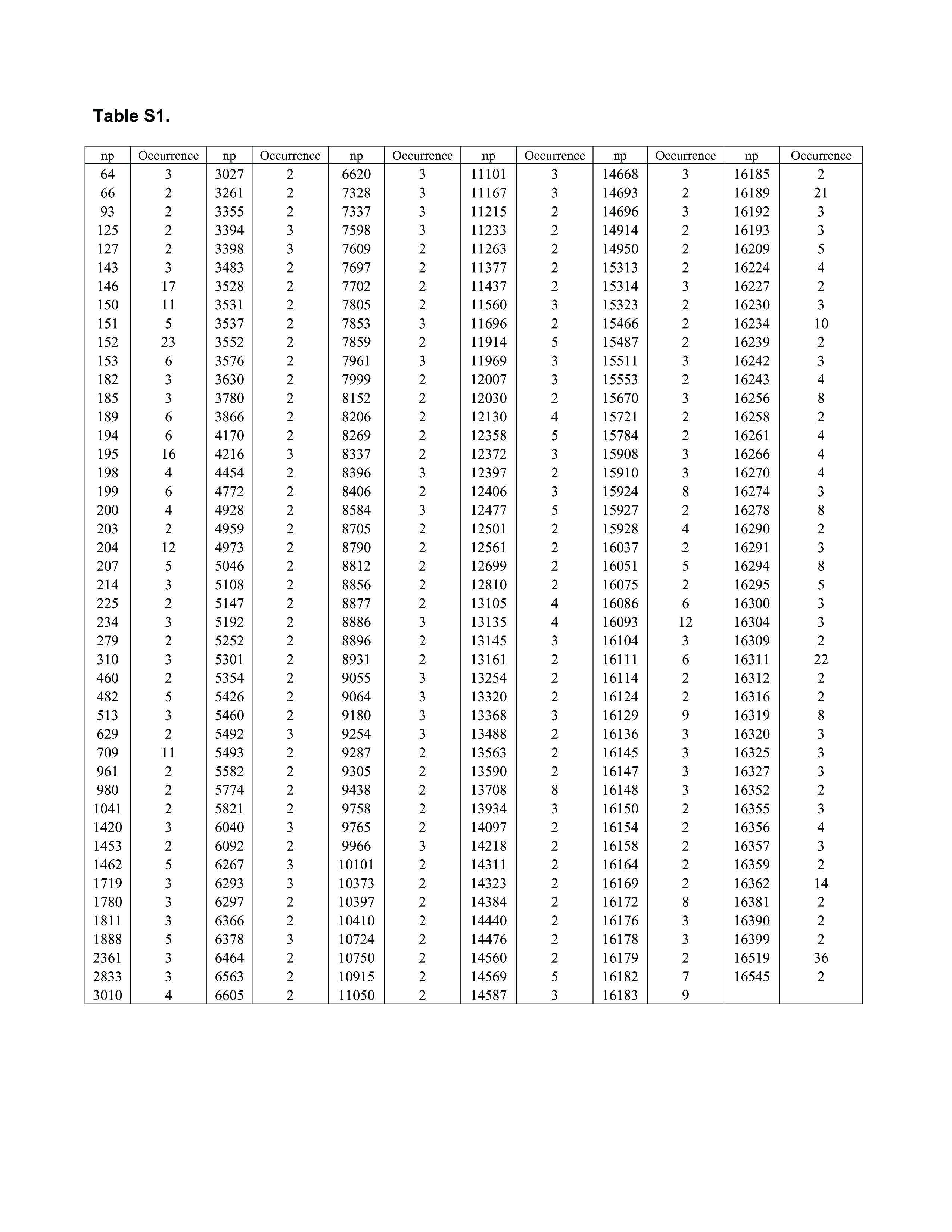

Supplement: Table S1 — Showing recurrence of mutations at various nucleotide positions (np). (4.33 MB TIF) [file pone.0007447.s001.tif]

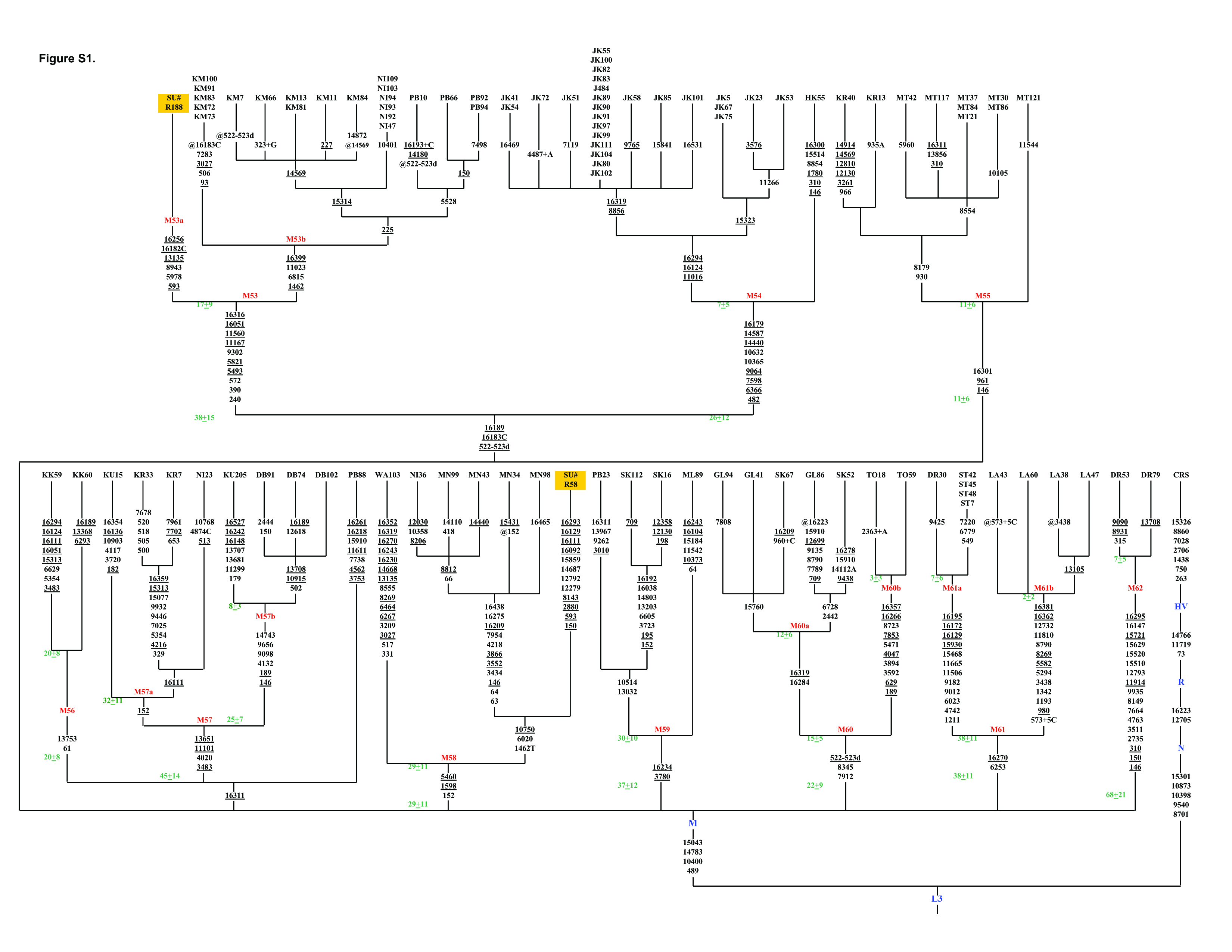

Supplement: Figure S1 — Indian mtDNA phylogenetic tree of macrohaplogroup M. Suffixes A, C, G, and T indicate transversions, “d” indicates a deletion, and a plus sign (+) indicates an insertion; 9bpins means 9-bp insertion (CCCCCTCTA) in the COII/tRNALys intergenic region. The A/C stretch length polymorphism in regions 16180–16193 and 303–315 and mutation 16519, all known to be hyper variable, were disregarded for tree reconstruction; recurrent mutations are underlined and the @ indicates back mutation. Samples code names were given in fig. 1.Samples collected from published sources were referred by symbols SU [22], TK [18], KG [11], TG [23], [43], KS [52], IG [3], BM [44], HE [2] and MC [48] followed by “#” and the original sample code. Haplogroup names indicated in Blue are defined in the earlier works, pink are redefined and red are newly identified in the present study. Coalescence times are based on synonymous mutation rate 3.5X10 ^-8 [52]. (3.76 MB TIF) [file pone.0007447.s002.tif]

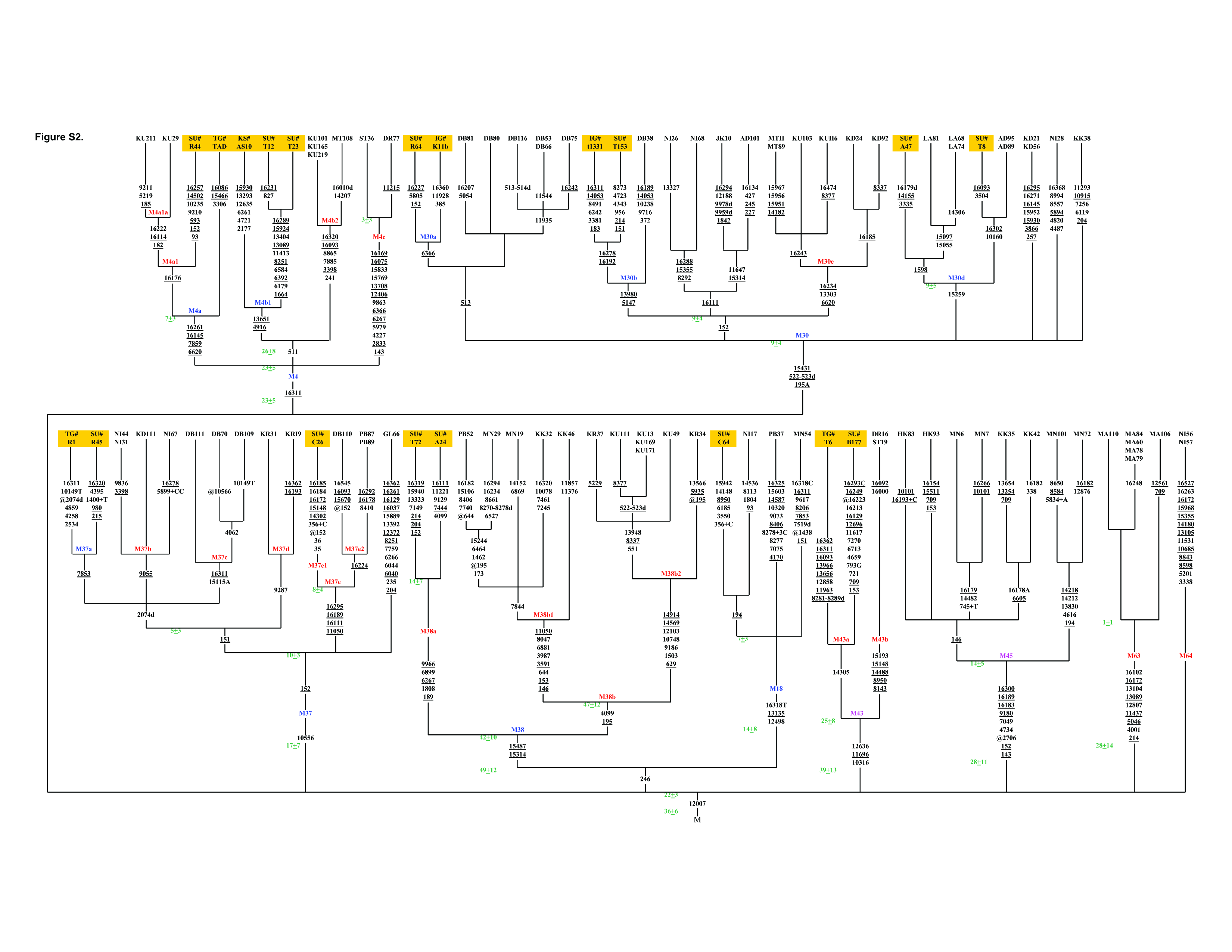

Supplement: Figure S2 — Indian mtDNA phylogenetic tree of macrohaplogroup M. Suffixes A, C, G, and T indicate transversions, “d” indicates a deletion, and a plus sign (+) indicates an insertion; 9bpins means 9-bp insertion (CCCCCTCTA) in the COII/tRNALys intergenic region. The A/C stretch length polymorphism in regions 16180–16193 and 303–315 and mutation 16519, all known to be hyper variable, were disregarded for tree reconstruction; recurrent mutations are underlined and the @ indicates back mutation. Samples code names were given in fig. 1.Samples collected from published sources were referred by symbols SU [22], TK [18], KG [11], TG [23], [43], KS [52], IG [3], BM [44], HE [2] and MC [48] followed by “#” and the original sample code. Haplogroup names indicated in Blue are defined in the earlier works, pink are redefined and red are newly identified in the present study. Coalescence times are based on synonymous mutation rate 3.5X10 ^-8 [52]. (3.85 MB TIF) [file pone.0007447.s003.tif]

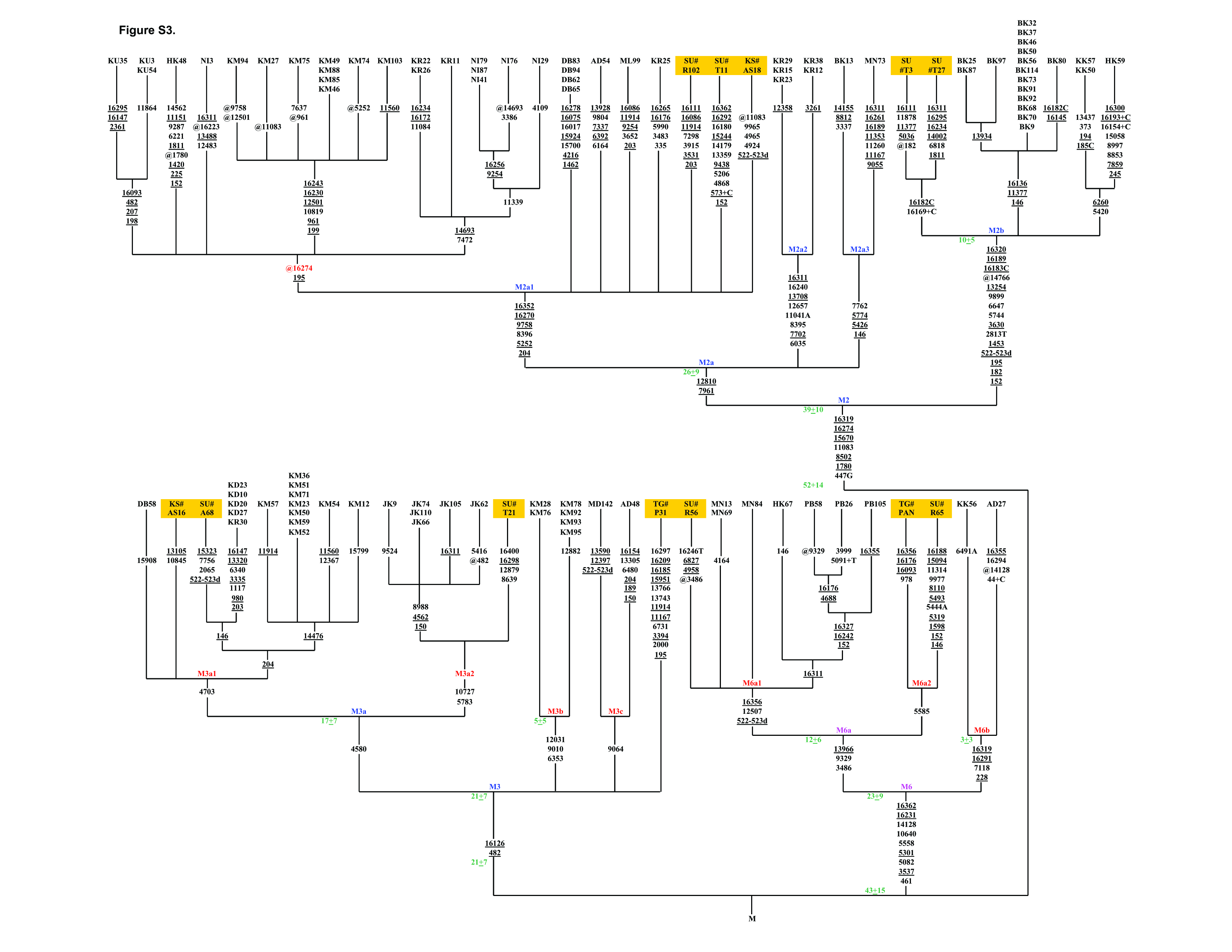

Supplement: Figure S3 — Indian mtDNA phylogenetic tree of macrohaplogroup M. Suffixes A, C, G, and T indicate transversions, “d” indicates a deletion, and a plus sign (+) indicates an insertion; 9bpins means 9-bp insertion (CCCCCTCTA) in the COII/tRNALys intergenic region. The A/C stretch length polymorphism in regions 16180–16193 and 303–315 and mutation 16519, all known to be hyper variable, were disregarded for tree reconstruction; recurrent mutations are underlined and the @ indicates back mutation. Samples code names were given in fig. 1.Samples collected from published sources were referred by symbols SU [22], TK [18], KG [11], TG [23], [43], KS [52], IG [3], BM [44], HE [2] and MC [48] followed by “#” and the original sample code. Haplogroup names indicated in Blue are defined in the earlier works, pink are redefined and red are newly identified in the present study. Coalescence times are based on synonymous mutation rate 3.5X10 ^-8 [52]. (3.69 MB TIF) [file pone.0007447.s004.tif]

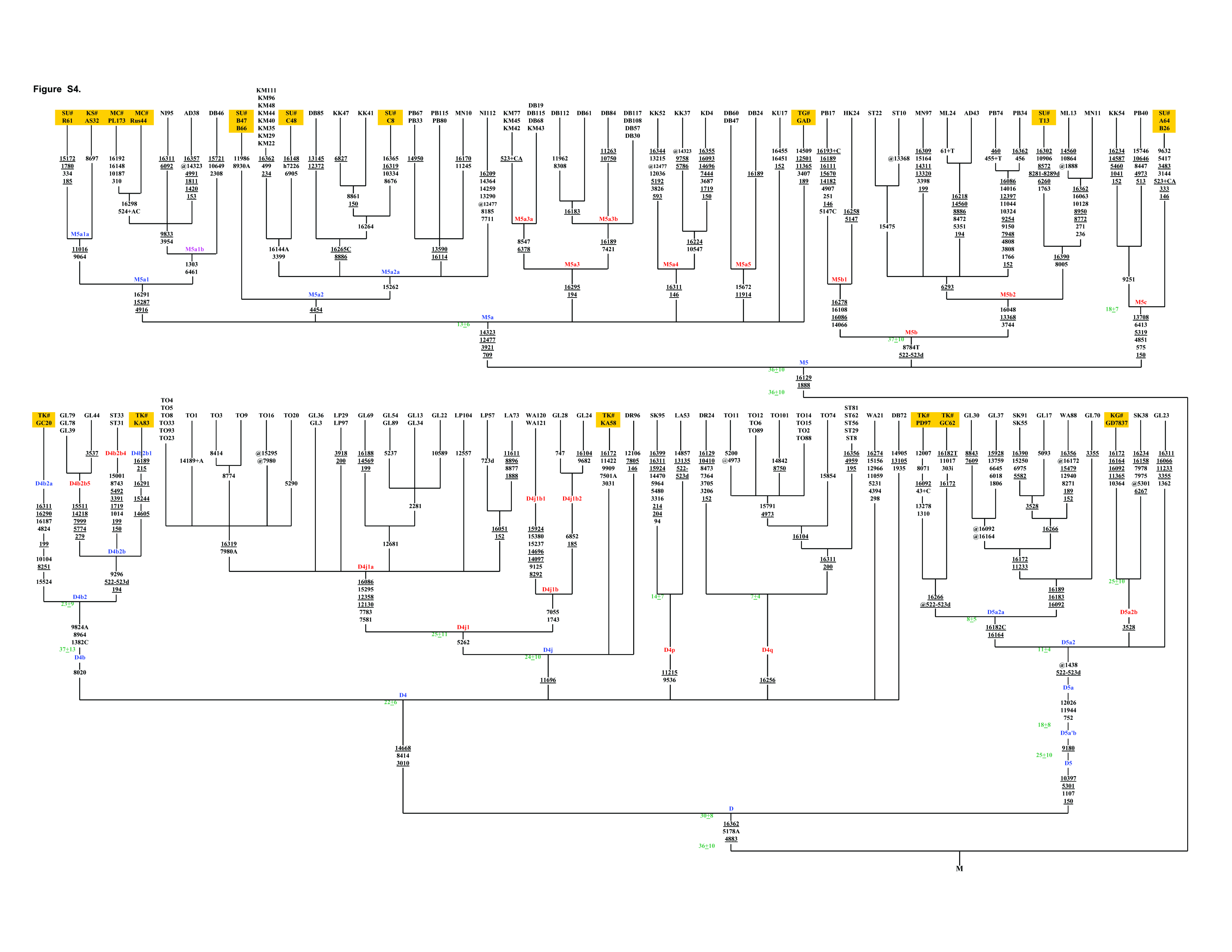

Supplement: Figure S4 — Indian mtDNA phylogenetic tree of macrohaplogroup M. Suffixes A, C, G, and T indicate transversions, “d” indicates a deletion, and a plus sign (+) indicates an insertion; 9bpins means 9-bp insertion (CCCCCTCTA) in the COII/tRNALys intergenic region. The A/C stretch length polymorphism in regions 16180–16193 and 303–315 and mutation 16519, all known to be hyper variable, were disregarded for tree reconstruction; recurrent mutations are underlined and the @ indicates back mutation. Samples code names were given in fig. 1.Samples collected from published sources were referred by symbols SU [22], TK [18], KG [11], TG [23], [43], KS [52], IG [3], BM [44], HE [2] and MC [48] followed by “#” and the original sample code. Haplogroup names indicated in Blue are defined in the earlier works, pink are redefined and red are newly identified in the present study. Coalescence times are based on synonymous mutation rate 3.5X10 ^-8 [52]. (3.94 MB TIF) [file pone.0007447.s005.tif]

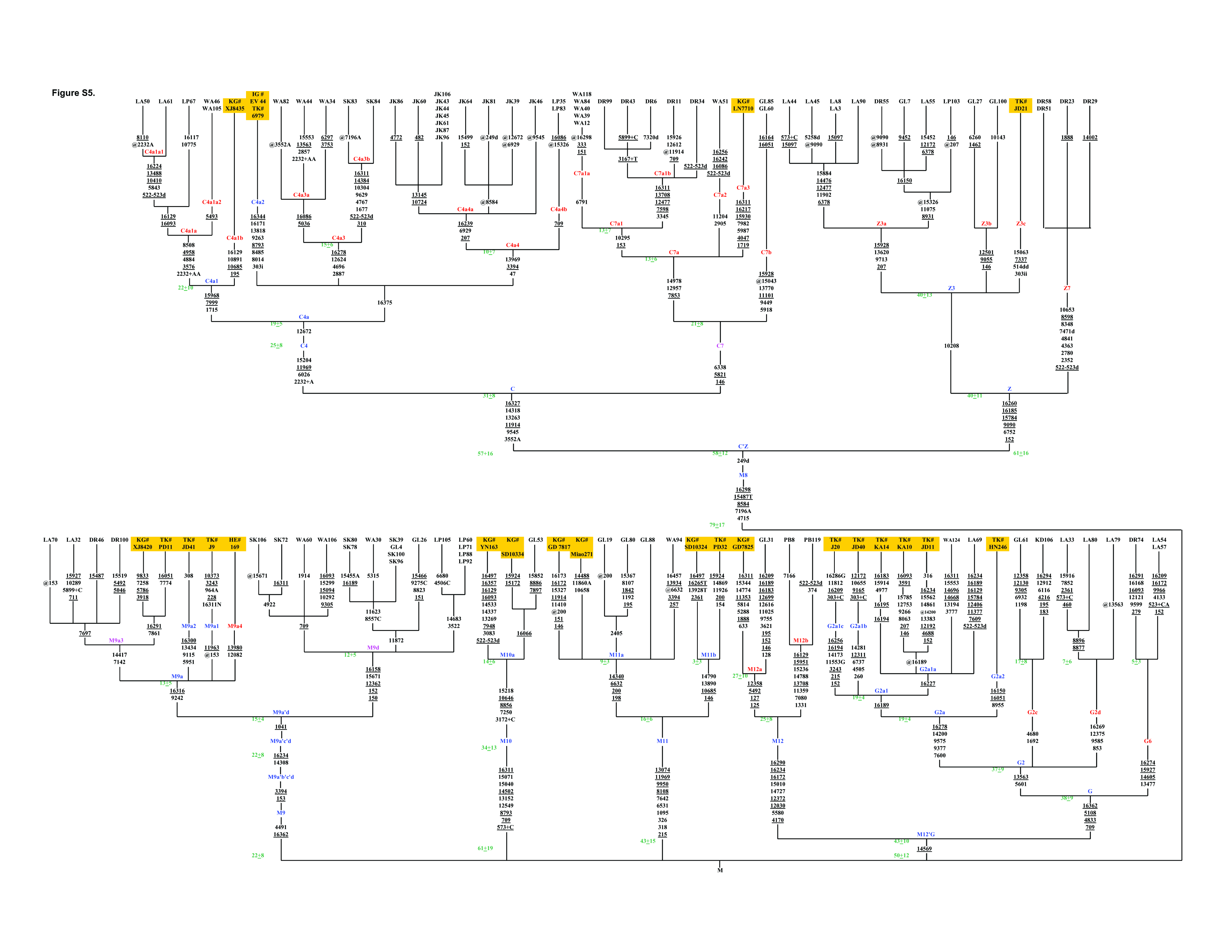

Supplement: Figure S5 — Indian mtDNA phylogenetic tree of macrohaplogroup M. Suffixes A, C, G, and T indicate transversions, “d” indicates a deletion, and a plus sign (+) indicates an insertion; 9bpins means 9-bp insertion (CCCCCTCTA) in the COII/tRNALys intergenic region. The A/C stretch length polymorphism in regions 16180–16193 and 303–315 and mutation 16519, all known to be hyper variable, were disregarded for tree reconstruction; recurrent mutations are underlined and the @ indicates back mutation. Samples code names were given in fig. 1.Samples collected from published sources were referred by symbols SU [22], TK [18], KG [11], TG [23], [43], KS [52], IG [3], BM [44], HE [2] and MC [48] followed by “#” and the original sample code. Haplogroup names indicated in Blue are defined in the earlier works, pink are redefined and red are newly identified in the present study. Coalescence times are based on synonymous mutation rate 3.5X10 ^-8 [52]. (3.84 MB TIF) [file pone.0007447.s006.tif]

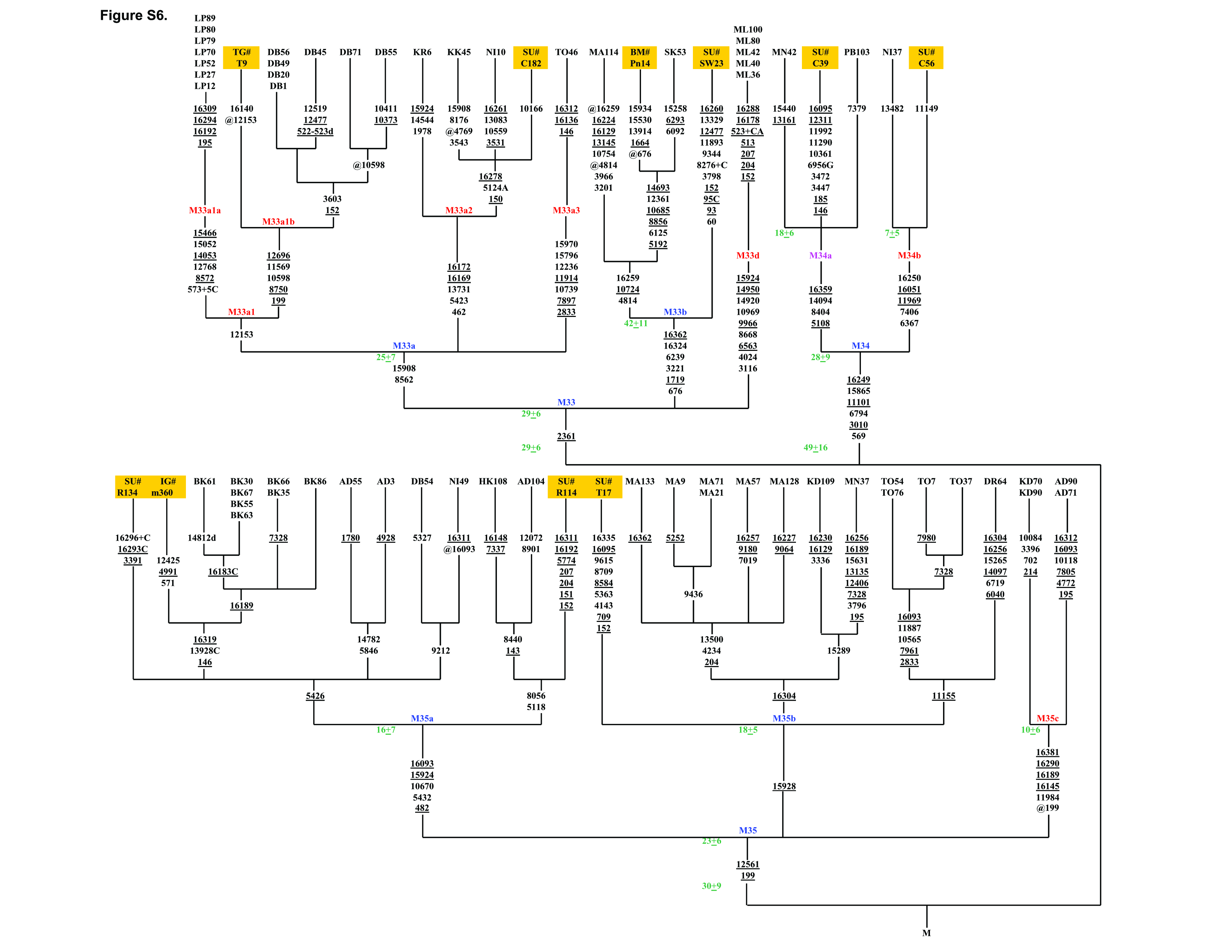

Supplement: Figure S6 — Indian mtDNA phylogenetic tree of macrohaplogroup M. Suffixes A, C, G, and T indicate transversions, “d” indicates a deletion, and a plus sign (+) indicates an insertion; 9bpins means 9-bp insertion (CCCCCTCTA) in the COII/tRNALys intergenic region. The A/C stretch length polymorphism in regions 16180–16193 and 303–315 and mutation 16519, all known to be hyper variable, were disregarded for tree reconstruction; recurrent mutations are underlined and the @ indicates back mutation. Samples code names were given in fig. 1.Samples collected from published sources were referred by symbols SU [22], TK [18], KG [11], TG [23], [43], KS [52], IG [3], BM [44], HE [2] and MC [48] followed by “#” and the original sample code. Haplogroup names indicated in Blue are defined in the earlier works, pink are redefined and red are newly identified in the present study. Coalescence times are based on synonymous mutation rate 3.5X10 ^-8 [52]. (3.57 MB TIF) [file pone.0007447.s007.tif]

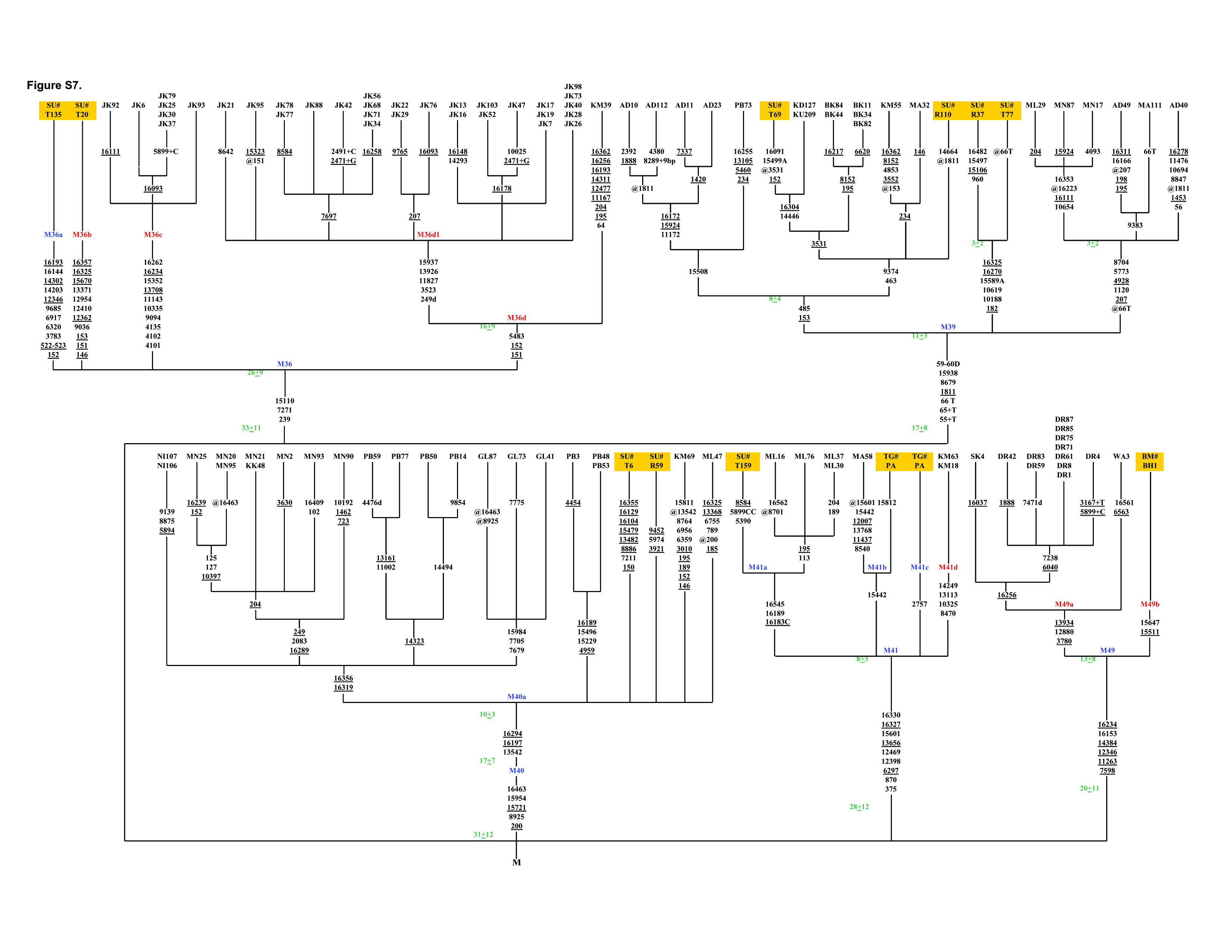

Supplement: Figure S7 — Indian mtDNA phylogenetic tree of macrohaplogroup M. Suffixes A, C, G, and T indicate transversions, “d” indicates a deletion, and a plus sign (+) indicates an insertion; 9bpins means 9-bp insertion (CCCCCTCTA) in the COII/tRNALys intergenic region. The A/C stretch length polymorphism in regions 16180–16193 and 303–315 and mutation 16519, all known to be hyper variable, were disregarded for tree reconstruction; recurrent mutations are underlined and the @ indicates back mutation. Samples code names were given in fig. 1.Samples collected from published sources were referred by symbols SU [22], TK [18], KG [11], TG [23], [43], KS [52], IG [3], BM [44], HE [2] and MC [48] followed by “#” and the original sample code. Haplogroup names indicated in Blue are defined in the earlier works, pink are redefined and red are newly identified in the present study. Coalescence times are based on synonymous mutation rate 3.5X10 ^-8 [52]. (3.59 MB TIF) [file pone.0007447.s008.tif]
